# Body mass index in girls with idiopathic central precocious puberty during and after treatment with GnRH analogues

**DOI:** 10.1186/s13633-016-0033-7

**Published:** 2016-08-05

**Authors:** A. J. Arcari, M. G. Gryngarten, A. V. Freire, M. G. Ballerini, M. G. Ropelato, I. Bergadá, M. E. Escobar

**Affiliations:** Centro de Investigaciones Endocrinológicas “Dr. César Bergadá” (CEDIE), CONICET – FEI – División de Endocrinología, Hospital de Niños Ricardo Gutiérrez, Gallo 1330, C1425EFD Buenos Aires, Argentina

**Keywords:** Central Precocious Puberty, BMI, GnRH analogues

## Abstract

**Background:**

In girls with Idiopathic Central Precocious Puberty (ICPP) concern has been raised by the potential impact of GnRH-analogues (GnRHa) treatment on body weight. We evaluated the effect of GnRHa on Body Mass Index (BMI) in girls with ICPP according to weight status at diagnosis.

**Methods:**

One hundred seventeen ICPP girls were divided according to pretreatment weight status in: normal weight (NW), overweight (OW) and obese (OB). BMI at one and two years of treatment was assessed. BMI-SDS of 60 patients who reached adult height (AH) was compared to that of 33 ICPP untreated girls.

**Results:**

NW girls significantly increased their baseline BMI-SDS at 1 and 2 years of treatment. OW girls only had a significant increment at one year of treatment while OB girls showed no BMI-SDS change. Patients evaluated at AH (at least four years after GnRHa withdrawal) showed a significant decrease on BMI compared to baseline and a significantly lower BMI than the untreated group.

**Conclusion:**

In ICPP girls the BMI increase under GnRHa was inversely related to the pretreatment weight status. In the long term follow-up, no detrimental effect of GnRHa on body weight was observed. BMI-SDS was lower in treated than in untreated girls.

## Background

Central Precocious Puberty (CPP) results from the premature activation of the hypothalamo-pituitary-ovarian axis. In girls, it is defined as the onset of secondary sexual development before the age of 8 with further progression, accompanied by increased growth velocity and bone age acceleration usually leading to adult height impairment.

Gonadotropin-releasing hormone analogues (GnRHa) are the treatment of choice in CPP and their effectiveness on adult height (AH) improvement has been widely recognized. However, concern has been raised by the potential impact of this treatment on body weight. While some studies have reported association between GnRHa treatment and Body Mass Index (BMI) increase [1–7], others have found no influence of GnRHa treatment on weight status [8–13] or even some decrease in BMI under therapy [14–16]. It has been demonstrated that girls with CPP are prone to developing obesity (8). A few reports have analyzed the influence of GnRHa on body weight according to BMI status at onset of treatment. However, in some of these studies follow-up was limited to a period under treatment or to a short time after withdrawal [17–19], whereas studies with a long-term follow-up did not categorize patients according to pre-treatment BMI status [20, 21].

The aim of the present study was to evaluate the impact of GnRHa therapy on BMI in girls with Idiopathic CPP (ICPP) according to weight status at diagnosis up to adult height (AH). The subgroup of patients who achieved AH was compared to a control group of untreated ICPP girls.

## Patients and methods

### Patients

This retrospective study was done reviewing the clinical charts of 117 girls with ICPP referred to the Division of Endocrinology of the Hospital de Niños Ricardo Gutiérrez (Buenos Aires, Argentina) from 1985 to 2010. All patients were treated for at least two years with intramuscular depot GnRHa (Triptorelin acetate), at a dose of 100–120 ug/kg, every 28 days for 2.8 ± 0.2 years; no patient received additional medications.

Inclusion criteria: patients with diagnosis of ICPP according to the following criteria: (1) onset of breast development before 8 years of chronological age (CA), (2) height velocity above the 97 centile for age and bone age (BA) advancement by at least one year over CA, (3) pubertal LH response to GnRH (≥ 6 mUI/ml), (4) uterine length ≥ to 35 mm. Exclusion criteria: organic central precocious puberty, congenital adrenal hyperplasia and any other underlying condition or medication that might affect body weight.

Patients were divided into three groups according to weight status at start of therapy: normal weight (NW); overweight (OW) and obese (OB). BMI at the end of first and second year of GnRH treatment was assessed. No specific life style intervention was recommended to any patient. At the time of reaching AH, auxological parameters in 60 patients were compared to those of a control group of 33 girls with ICPP who had not received treatment (age of menarche 9.5 ± 0.3).

## Methods

Clinical and auxological features were assessed as follows: pubertal stage according to Marshall and Tanner criteria [22]; height in cm measured by a Harpenden stadiometer; weight using a calibrated scale; bone age according to Greulich and Pyle [23].

Weight status was determined as BMI and it was expressed as BMI-SDS using the software Auxology version 1.0 b 17 Copyright® 2003 Pfizer. Overweight was defined as BMI > 85^th^ centile and obesity as BMI > 95^th^ centile according to Cole criteria [24].

AH was defined when two successive height measurements six months apart were equal or less than 0.5 cm, and/or when BA was equal or greater than 15 years. This study fulfilled the requirements defined by the Ethical Committee of Hospital de Niños Ricardo Gutiérrez for retrospective studies. Reference number CEI 11.053.

Statistical analyses were performed using GraphPad Prism version 5.00 for Windows (GraphPad Software, San Diego, CA). Changes on BMI were analyzed by ANOVA for repeated measurements. *Post hoc* Tukey’s multiple comparison test was performed when differences were detected by ANOVA. Chi square test was used to compare the percentage of NW, OW and OB girls in the whole group at treatment start vs the percentage of NW, OW and OB girls in those who reached AH. The comparison between patients and control girls who reached AH was done using Mann Whitney test. *P* < 0.05 was considered statistically significant. Data is expressed as mean ± SD.

## Results

A total of 117 girls with ICPP were studied. At treatment start all patients had breast development Grade II-III (Tanner) and three of them have had menarche. CA was 7.6 ± 1.3 years, BA 9.6 ± 0.16 years, height SDS 1.71 ± 0.08. The uterine length was 37.2 ± 0.7 mm.

Before treatment, BMI in the whole group was 18.5 ± 2.4 (SDS 1.1 ± 1). BMI distribution by groups showed: 48 % girls NW, 37 % OW and 15 % OB. No significant difference in chronological age was observed among groups, p: ns (Table 1).

**Table 1:** Auxological characteristics of the whole group at start of treatment

	Age (years)	BMI at start (kg/m^2^)	BMI-SDS at start
All patients (*n* = 117)	7.6 ± 1.3	18.5 ± 2.4	1.1 ± 1
Normal Weight (*n* = 56)	7.53 ± 1.3	16.5 ± 1.3	0.3 ± 0.7
Overweight (*n* = 43)	7.33 ± 1.5	19.3 ± 4.7	1.5 ± 0.3
Obese (*n* = 18)	7.74 ± 0.8	22.4 ± 1.2	2.4 ± 0.3

**Fig. 1:**
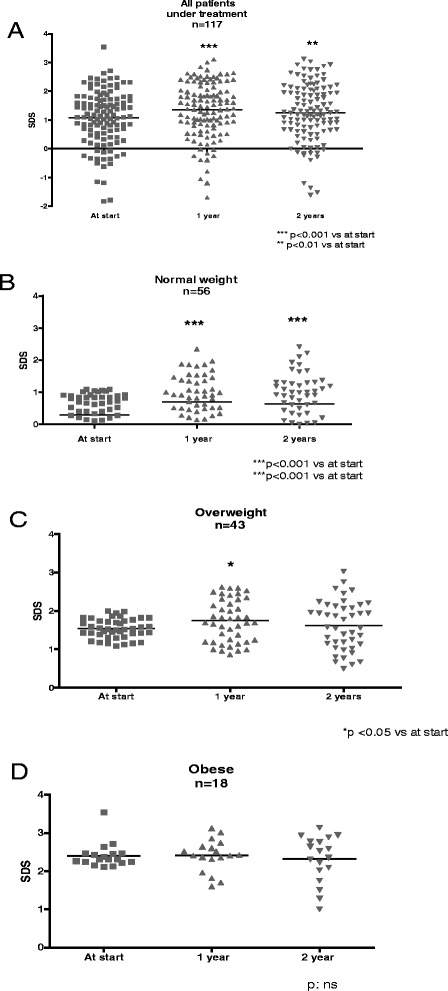
BMI evolution of patients with ICPP during treatment with GnRHa. **a** All, **b** Normal weight, **c** Overweight, **d** Obese

The whole cohort of patients significantly increased their baseline BMI-SDS (1.1 ± 1) at year one (1.35 ± 0.95, *p* < 0.001) and year two (1.26 ± 0.1 *p* < 0.01) of treatment. When changes in BMI were analyzed according to the initial weight status, NW patients showed a significant increase in BMI-SDS from 0.3 ± 0.7 to 0.7 ± 0.8 at one year of treatment and 0.6 ± 0.8 at two years (*p* < 0.001). In OW girls a significant increase was only observed between baseline (1.5 ± 0.2) and one year of treatment (1.7 ± 0.5) (*p* < 0.05). OB girls showed no BMI-SDS changes during treatment (baseline 2.4 ± 0.3, year one 2.4 ± 0.4 and year two 2.3 ± 0.6) (Fig. 1).

**Fig. 2:**
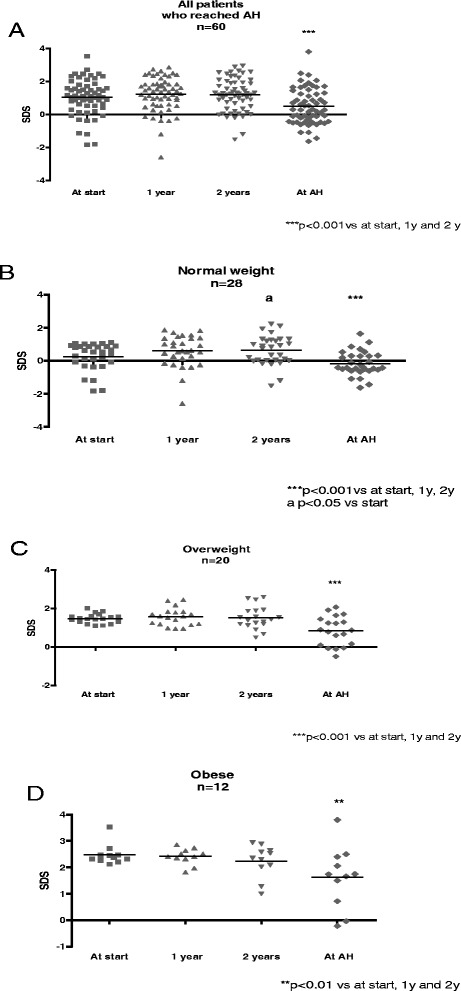
BMI evolution during treatment in 60 patients at the time of reaching Adult Height. **a** All, **b** Normal weight, **c** Overweight, **d** Obese

**Fig. 3:**
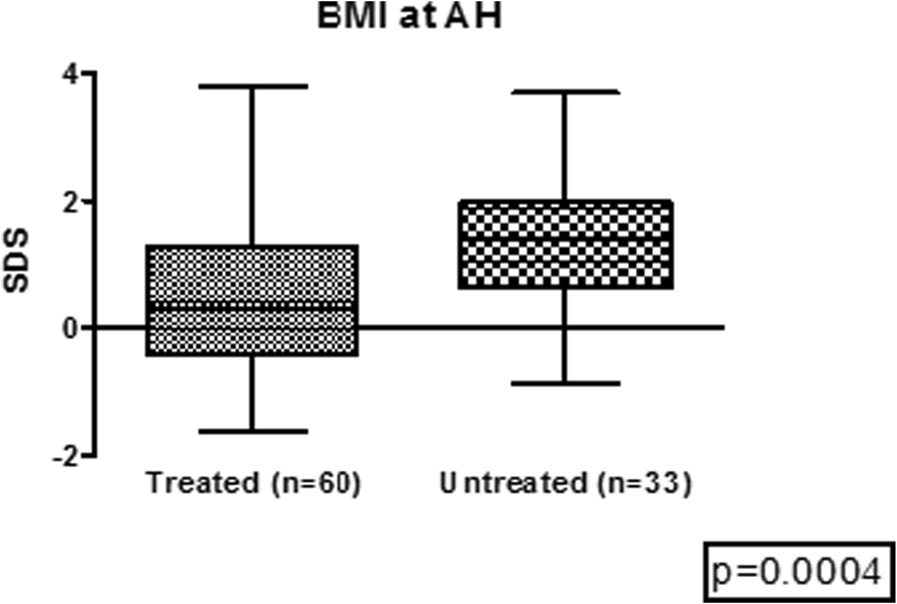
BMI at adult height treated vs. untreated patients

A subset of 60 girls were followed up to AH [160.4 ± 0.7 cm (SDS −0.01 ± 0.14)], which was achieved at 4.4 ± 0.4 years after GnRHa withdrawal. BMI composition at beginning of treatment of the group of girls who attained AH was not different from that of the whole group (NW 47 % vs 48 %, OW 33 % vs 37 %, OB 20 % vs 15 % respectively, *p* = ns). All girls in the AH group showed a significant decrease on BMI-SDS at the moment of reaching AH (0.5 ± 1.1) compared to BMI-SDS at start (1.0 ± 1.1), *p* < 0.001. A similar pattern was observed when the BMI status was analyzed in each subgroup (Table 2) (Fig. 2). The BMI-SDS of the 60 treated patients who reached adult height was 0.5 ± 1.1, significantly lower than the BMI-SDS of the untreated control group, 1.3 ± 1.00 (*p* = 0.0004) (Fig. 3).

**Table 2:** Auxological characteristics in girls who reached adult height (AH)

	BMI at start (SDS)	BMI at AH (SDS)
All patients (*n* = 60)	1.0 ± 1.1	0.5 ± 1.1
Normal Weight (*n* = 28)	0.25 ± 0.8	−0.19 ± 0.7
Overweight (*n* = 20)	1.4 ± 0.2	0.7 ± 0.7 b,c
Obese (*n* = 12)	2.4 ± 0.4	1.57 ± 1.1 a

ANOVA a < 0.0001 vs NW, b 0.001 vs NW, c 0.03 vs OB. Unpaired *t* Test start vs at AH: All patients *p* 0.004, NW *p* 0.07, OW *p* 0.0004, OB *p* 0.03

The evolution of the weight status from start to AH in each category is shown in Table 3.

**Table 3:** Evolution of BMI category during follow-up in each subgroup

At start	At Adult Height (N° of patients)
NW *n* = 28	NW 27
	OW 1
	OB 0
OW *n* = 20	NW 14
	OW 5
	OB 1
OB *n* = 12	NW 4
	OW 4
	OB 4

## Discussion

The present study evaluates the long-term effect of a monthly depot GnRHa therapy on BMI in a large cohort of 117 girls with ICPP from a single endocrine pediatric center in relation to weight status at diagnosis, along treatment and at AH in a subset of 60 patients.

Girls with ICPP at the moment of diagnosis usually have a high prevalence of overweight and obesity [17–19, 25], although if early puberty is the cause or the consequence of the increased body fat remains unclear. In agreement with the literature, before treatment our patients had an elevated proportion of overweight and obesity, 37 and 15 % respectively, these rates are higher than those of average Argentinian girls of the same age, 20.8 and 5.4 % [26].

The impact of GnRHa treatment on body weight in our patients was clearly related to the pretreatment condition. Although the whole cohort of ICPP patients showed a BMI increase along treatment, the negative effect of the GnRHa seemed to decrease as the pretreatment weight was higher. Girls in the NW subgroup showed an increase of BMI at one and two years of treatment while OW girls had a higher BMI only at year one, while no changes were observed in OB girls along the treatment.

The inconsistency in the results about the effect of GnRHa on body weight found in the literature could be related to differences in the studied populations, i.e., mixed early and precocious puberty, idiopathic and organic CPP, and/or comparison with heterogeneous control groups.

While some authors found no changes in body weight on ICPP patients under GnRHa [8–13], few studies categorized patients according to pretreatment BMI to analyze the effect of treatment. Some reports showed that OW/OB girls at baseline remained unchanged or increased at the end of treatment, without any reference to NW patients [27, 28].

Lee et al. studied a group of girls with CPP (including 3 with organic CPP) during 18 months on treatment with GnRHa, and showed a significant BMI increase in the whole group, higher although not significant, in NW than in OW patients [18].

Wolters et al. evaluated a mixed group of girls with early or precocious puberty treated with GnRHa during one year according to pretreatment weight status and compared the OW subgroup to an OW untreated control group. In agreement with our study, their whole population of girls with CPP had a significant BMI increase along treatment, as well as the NW subset patients, whereas OW girls showed no changes in BMI while the OW control group showed a BMI increase [17].

Colmenares et al. analyzed 37 girls with ICPP and 34 girls with early puberty under GnRHa treatment, compared to a heterogeneous control group of untreated girls (3 with CPP who refused treatment, 2 with slowly progressive CPP and 20 with early slowly progressive puberty). In this study 72.9 % of the girls with ICPP were OW or OB, a percentage significantly higher than that of OW/OB girls with early puberty (35.3 %). The entire group of treated ICPP girls showed no changes in BMI at one and two years of treatment, whereas an increase at year three was observed compared to the control group. However, no individual analysis was performed in relation to the weight status at baseline [19].

The few reports evaluating body weight status in ICPP patients after GnRHa treatment showed return to pretreatment values [27] or no differences in BMI when compared to untreated control girls [20, 29]. Lazar et al. analyzed the evolution of a large cohort of CPP treated patients at the third to fifth decades of life and found that although the BMI was increased at start and during early treatment, it normalized thereafter [30]. None of the studies with a long-term follow-up analyzed BMI in relation to the weight status at the beginning of treatment.

Our subgroup of 60 patients evaluated at least four years after GnRHa withdrawal, when they had reached AH, showed a significant decrease in BMI compared to pretreatment BMI. The same pattern was observed when BMI status was analyzed for each subgroup. Approximately 2/3 of OW and 2/3 of OB patients showed a decrease in weight status, whereas only 1 /28 NW girls had become OW at AH. Furthermore, this subgroup with a long-term follow-up had a significantly lower BMI than the control group of untreated CPP girls, both compared at AH.

Categorizing our patients according to their weight status at start, along and after treatment allowed us to observe that only NW girls increased their BMI during treatment, while OW and OB girls did not, despite no changes in dietary habits or exercise were prescribed. Wolters et al. found a similar BMI behavior in CPP treated patients. The BMI of their OW girls (authors did not categorize between OW and OB) remained stable while a control group of untreated girls showed a significant BMI increase during follow-up, and they suggested a positive effect of GnRHa on weight status in OW children [17]. We did not have a control group of untreated patients evaluated as from the moment of diagnosis, but the comparison between our treated CPP girls with our group of untreated patients at AH status showed a significantly lower BMI among treated girls, thus seeming to confirm Wolters´s suggestion.

A limitation in our study was that we were able to analyze the body composition as a marker of nutritional status only on a small group of girls. There are a few reports evaluating the changes in body composition in CPP under GnRHa treatment. Van der Sluis et al. observed an increase of fat mass (estimated by DEXA) during treatment and a normalization after withdrawal. Chiocca et al. analyzed a cohort of CPP girls after GnRHa treatment at near final height and found no changes in BMI but an increase of total fat mass compared to a control group. Both authors suggested that the body fat accumulation was related to the estrogen depletion produced by GnRHa, as a “menopausal effect” [5, 14].

At present, there is no clear explanation of the different effects produced by GnRHa treatment on lean and overweight ICPP girls. Although no specific prescription on life style was given to our patients, it is not possible to rule out that the fact that OW/OB girls did not increase their BMI under treatment could have been related to a family concern about weight gain leading to changes in dietary or physical activity.

Although in the present study, we did not systematically analyze the metabolic profile of our patients, Colmenares et al. found no differences for glucose and lipids at baseline and during follow-up among treated children [19].

## Conclusions

In conclusion, in our study, although a moderate increase in BMI was observed throughout the first and second year on GnRHa treatment, mainly in NW girls, such increase did not lead to further obesity since a significant improvement on BMI was observed when girls achieved adult height. Furthermore, the fact that treated girls had a lower BMI than untreated girls at adult height allowed us to rule out a long-term detrimental effect of GnRHa treatment on body mass in girls with Central Precocious Puberty. Further studies analyzing changes in body composition are desired.

## Abbreviations

AH, adult height; BA, bone age; BMI, Body Mass Index; CA, chronological age; CPP, Central Precocious Puberty; GnRHa, GnRH-analogues; ICPP, Idiopathic Central Precocious Puberty; NW, normal weight; OB, obese; OW, overweight
